# Microglia and infiltrating macrophages in ictogenesis and epileptogenesis

**DOI:** 10.3389/fnmol.2024.1404022

**Published:** 2024-05-30

**Authors:** Sonja Bröer, Alberto Pauletti

**Affiliations:** Institute of Pharmacology and Toxicology, School of Veterinary Medicine, Freie Universität Berlin, Berlin, Germany

**Keywords:** macrophage, microglia, inflammation, seizure, phagocytes, epileptogenesis, innate immune cells

## Abstract

Phagocytes maintain homeostasis in a healthy brain. Upon injury, they are essential for repairing damaged tissue, recruiting other immune cells, and releasing cytokines as the first line of defense. However, there seems to be a delicate balance between the beneficial and detrimental effects of their activation in a seizing brain. Blocking the infiltration of peripheral phagocytes (macrophages) or their depletion can partially alleviate epileptic seizures and prevent the death of neurons in experimental models of epilepsy. However, the depletion of resident phagocytes in the brain (microglia) can aggravate disease outcomes. This review describes the role of resident microglia and peripheral infiltrating monocytes in animal models of acutely triggered seizures and epilepsy. Understanding the roles of phagocytes in ictogenesis and the time course of their activation and involvement in epileptogenesis and disease progression can offer us new biomarkers to identify patients at risk of developing epilepsy after a brain insult, as well as provide novel therapeutic targets for treating epilepsy.

## 1 Introduction

The birth of microglial research dates back more than one hundred years ago to Pío del Río-Hortega’s pioneering work (Hortega et al., 1919), identifying microglia as mutable and plastic cells of the central nervous system (CNS). In physiological states, phagocytes are essential for brain maturation, immune surveillance, debris clearance, and synaptic pruning, contributing to the maintenance of CNS homeostasis. Conversely, in pathological states, their activation, as well as their dysregulation, are implicated in a spectrum of CNS disorders, including acute and chronic seizures. There seems to be a delicate balance between their activation’s beneficial and detrimental effects in a seizing brain. This review describes the role of resident microglia and peripheral infiltrating monocytes in animal models of acutely triggered seizures and epilepsy, and in human patients with seizures. Many other innate and adaptive immune cells are involved in inflammation, or even serve as activators of monocytes in the very early stages of inflammation, e.g., reactive astrocytes. Our review however highlights the role of microglia and macrophages in icto- and epileptogenesis, and the role of astrocytes is discussed elsewhere (Heuser et al., 2021; Li et al., 2023).

## 2 Physiological role of phagocytes in brain homeostasis

The maintenance and immune surveillance of the CNS are managed by a sophisticated cellular framework of resident microglia, comprised of parenchymal microglia and extra-parenchymal CNS-associated macrophages (CAMs) (Ginhoux et al., 2010; Goldmann et al., 2016). Both cell types originate from the embryonic yolk sac (Ginhoux et al., 2010, Goldmann et al., 2016), and share transcriptomic signatures, such as the expression of ionized calcium-binding adaptor molecule 1 (Iba1), fractalkine receptor (Cx3cr1), and colony-stimulating factor 1 receptor (Csf1r). These markers indicate the fundamental immune capabilities of these cells within the CNS (Zeisel et al., 2015; Goldmann et al., 2016; Jordão et al., 2019). Nevertheless, as different cell populations, they also express specific markers that make them able to perform their unique functions, for example, P2Y purinergic receptor 12 (P2ry12), and transmembrane protein 119 (Tmem119) for microglia and mannose receptor 1 (Mrc1 or CD206) for CAMs (Zeisel et al., 2015; Goldmann et al., 2016; Jordão et al., 2019).

Microglia are necessary for brain development and homeostatic maintenance. Microglia shape adult neurogenesis (Sierra et al., 2010), promote synapse maturation and plasticity (Paolicelli et al., 2011), remodel neuronal circuits (Paolicelli et al., 2011; Hoshiko et al., 2012; Ueno et al., 2013; Frost and Schafer, 2016), the development of oligodendrocyte progenitors, and the subsequent myelination process (Hagemeyer et al., 2017). In a healthy adult CNS, microglia show a “surveying/resting” phenotype characterized by the dynamic reconfiguration of their processes (Hanisch and Kettenmann, 2007). When engulfing apoptotic cells or myelin debris, anti-inflammatory factors, such as prostaglandin E2 and reactive oxygen species (ROS) are released in microglia, and anti-inflammatory cytokines, notably interleukin-10 (IL-10), are produced (Hanisch and Kettenmann, 2007). Microglia activation is a functional phenotype transformation from their surveying/resting state, which occurs after they sense several specific signals, such as noxious stimuli. Such recognition disrupts the “off” signal, triggering an alert and activation response (Hanisch and Kettenmann, 2007). Upon activation, microglial cells can adopt various response phenotypes. For example, in the presence of bacterial invasion, microglia engage in phagocytosis and release pro-inflammatory mediators, including tumor necrosis factor-alpha (TNF-α), interleukin-6 (IL-6), interleukin-12 (IL-12), keratinocyte chemoattractant (KC), monocyte chemoattractant protein-1 (MCP-1), macrophage inflammatory protein-1α (MIP-1α), macrophage inflammatory protein-2 (MIP-2), and regulated on activation, normal T cell expressed and secreted (RANTES), as well as soluble TNF receptor II, which acts as a potential antagonist of TNF-α (Häusler et al., 2002). Similarly, CAMs are specialized macrophages at the CNS barriers and are involved in early pathogen detection and danger signaling by releasing cytokines and chemoattractants as recruitment for other immune cells (Kierdorf et al., 2019).

Monocytes, pivotal components of the innate immune system, constitute a fraction of the myeloid lineage cells, originating from hematopoietic stem cells within the bone marrow matrix. They are found in the spleen, bone marrow, and bloodstream, respond to both inflammatory and pathogenic stimuli, and maintain homeostasis by transitioning into tissue-infiltrating macrophages (Ashhurst et al., 2014; Italiani and Boraschi, 2014). Depending on the required activity, they can differentiate into two distinct classes of macrophages: M1 or “inflammatory” macrophages (CD11b + CD45hi Ly-6Chi) and M2 or “anti-inflammatory/patrolling” macrophages (CD11b + CD45hi Ly-6Clow) (Ashhurst et al., 2014; Italiani and Boraschi, 2014; Katsumoto et al., 2014). This distinction is also underscored by their secretion of specific molecules defining their function: (1) pro-inflammatory interleukins (IL-1β, TNF-α, IL-6, and inducible nitric oxide synthase (iNOS)) are released by M1 macrophages to amplify inflammation, and they express high levels of the chemokine receptor CCR2. CCR2 facilitates the invasion of monocytes to the CNS, and binds to CCL2, a ligand expressed by various CNS cells after injury or infection (Bosco et al., 2020). (2) On the contrary, immunosuppressive cytokines, such as IL-10 are secreted by M2 macrophages to mitigate inflammation and restore homeostasis. They express higher levels of the fractalkine receptor (CX3CR1) (Italiani and Boraschi, 2014). In a healthy brain, monocytes are predominantly confined to the cerebral vasculature, within the dura mater, where they serve for the turnover of CAMs (Mundt et al., 2022) and are seldom encountered in the CNS parenchyma (De Vlaminck et al., 2022). Nevertheless, CNS monocytes are equipped with pathogen-recognition receptors (PRRs) and might represent the first sentinels of CNS infection or insult (Rua et al., 2019), including seizures.

## 3 Phagocytes in epileptogenesis

### 3.1 Epileptogenesis

The word epileptogenesis describes the process during which a healthy brain is transformed into an epileptic brain with chronic, spontaneously recurring seizures, as well as the progression of the disease once chronic seizures have started (Pitkänen et al., 2015). Epileptogenesis is triggered by an initiating brain insult (e.g., trauma, stroke, infection; Ravizza et al., 2008; Löscher and Brandt, 2010). While the initiating insult itself can produce acute seizures, the subsequent process of epileptogenesis typically is accompanied by a seizure-free latent period. Depending on individual and insult-specific factors, this developmental period can last weeks, months or years in human patients (White and Löscher, 2014). In animal models of epilepsy, it is mainly completed within days to weeks. Depending on the severity of the insult, the age of the patient, and any genetic predispositions, the insult may produce several alterations in brain homeostasis. Many findings from experimental models and also from human patients point to a key role for inflammation, specifically to resident and infiltrating monocytes in insult-associated seizures, status epilepticus (SE), and epileptogenesis.

### 3.2 Clinical evidence for phagocyte involvement

Data from resected brain tissue and post-mortem exams of human brains show that activated microglia, astrocytes, and in some cases also migrated leukocytes, in particular macrophages, neutrophil granulocytes, or T-lymphocytes are present in the brain parenchyma in epilepsy (Vezzani et al., 2011, 2016). Indeed, many autoimmune diseases are associated with the occurrence of seizures, for example, multiple sclerosis, systemic lupus erythematosus (Najjar et al., 2008), and a number of autoantibodies are associated with autoimmune epilepsy (Husari and Dubey, 2019). Other causes of encephalitis are infections. Cytokines that are increased intracerebrally during fever can lead to increased seizure susceptibility (Dubé et al., 2007, 2010). Up to 30% of patients with a CNS infection suffer from symptomatic seizures (Vezzani et al., 2016). Approximately 20% of patients who survive a viral CNS infection develop epilepsy (Annegers et al., 1988). Herpes simplex virus type-1 (HSV-1), non-polio picornaviruses, Zika virus (ZIKV), West Nile virus (WNV), Japanese encephalitis virus, cytomegalovirus, human herpes virus-6 and recently SARS-CoV-2 have been described to be able to trigger symptomatic seizures (Solomon et al., 2000; Suzuki et al., 2008; Bartolini et al., 2019; Ellul et al., 2020; DePaula-Silva et al., 2021).

Inflammatory processes are not only present in epilepsies with apparent immune system involvement. In 2002, Crespel and colleagues reported that reactive astrocytes and neurons of lesioned areas in surgically resected hippocampi from patients who have mesial temporal lobe epilepsy (TLE) and hippocampal sclerosis over-expressed transcription factor NFκB, which was not found in control hippocampi without epilepsy (Crespel et al., 2002). NFκB regulates genes involved in the immune response. Furthermore, pro-inflammatory cytokines (IL-1β, IL-6, TNF-α), molecules of the complement system, inflammation-associated proteins like HMGB1 or receptors involved in inflammation (TLR4, IL-1 receptor type 1), as well as monocytes were found in patients (Ravizza et al., 2008; Vezzani et al., 2016; Vezzani, 2020). Imaging the binding of translocator protein 18 kDa (TSPO), a biomarker of neuroinflammation, Gershen et al. (2015) and Dickstein et al. (2019) were able to confirm that ongoing inflammation was present during interictal periods in patients with TLE and neocortical epilepsy.

#### 3.2.1 Microglia involvement in clinical epilepsy

Investigation of resected tissue from patients with epilepsy showed a high number of activated and proliferating microglia in the hippocampus versus the resting, ramified microglia in epilepsy-unrelated autopsy cases (Beach et al., 1995; Najjar et al., 2011). CX3CL1 expression was upregulated in brain tissue, cerebrospinal fluid, and serum of patients with epilepsy (Xu et al., 2012; Roseti et al., 2013). CXCL1 is involved in many neuroinflammatory conditions by facilitating neuron–microglia interactions, including induced cell death via the CXCR1 receptor on the surface of microglia (Kastenbauer et al., 2003; Ransohoff, 2009). Another proof for the involvement of microglia in epilepsy-associated cell death was found by Altmann and colleagues, who performed a systems-level analysis of large datasets of neuroimaging, GWAS, and post-mortem tissue of patients with epilepsy to identify commonalities in cortical thinning, a structural consequence of epilepsy: Activated microglia and their genes were highly expressed in the thinned cortical areas (Altmann et al., 2022). Another elegant approach using single-cell transcriptomics and surface epitope detection of immune cells from surgically resected human epileptic brain tissues confirmed pro-inflammatory signaling in microglia, with high expression levels of the pro-inflammatory genes IL1B, IL18, CXCL8 (IL-8) and CCL4 (Kumar et al., 2022). A majority of clusters were identified as microglia (CD45lo), but some were recognized as infiltrating immune cells (CD45hi; Kumar et al., 2022).

#### 3.2.2 Macrophage involvement in clinical epilepsy

CNS recruitment of macrophages is induced by increased CCL2 expression, mainly by damaged neurons, e.g., after SE. In healthy control brain tissue, only low numbers of inactive macrophages were present around the blood vessels, while post-mortem samples from patients that died during SE as well as chronic seizure patients showed higher CCL2 expression, and significantly more and activated macrophages were found throughout the brain parenchyma (Broekaart et al., 2018). Recently, Charles Howe reported intriguing findings in a pediatric patient with seizures on the involvement of peripheral monocytes in a febrile infection-related epilepsy syndrome (FIRES) (Howe et al., 2023): (1) Many inflammatory markers were upregulated in the peripheral blood during SE. (2) Upon *ex vivo* bacterial stimulation with LPS, isolated peripheral blood mononuclear cells (PMBCs) from the pediatric patient produced a strong release of IL-6 and CXCL8. (3) Inflammatory responses and refractory seizures resolved after several intrathecal injections of dexamethasone, an anti-inflammatory glucocorticoid. These data prompted the group to postulate that FIRES might be linked to an exaggerated, unfavorable pro-inflammatory response of peripheral monocytes to rather banal bacterial infections. This data supports experimental evidence from Howe’s preclinical studies, our group, and others for the pivotal part that peripheral monocytes invading the CNS could play in many acute and chronic seizures (see below).

Despite evidence for the involvement of microglia and macrophages in human epilepsy and novel methods, such as transcriptomics and proteomics to elaborately measure the presence and functional state of these cells, our current knowledge on neuroinflammation stems primarily from preclinical data, and still little is known about human phagocyte biology (Prinz et al., 2019).

### 3.3 Experimental evidence for phagocyte involvement

Inflammation has been characterized across various rodent models of seizures and epilepsy. Most epilepsy models use SE as an initial insult to induce epileptogenesis, either by electrical stimulation or the application of proconvulsant substances (Löscher, 2011). Despite the different induction, it is uniformly described that SE leads to increased microglial activation. Microglia change their shape, and release several pro-inflammatory cytokines quickly after seizures start (Beach et al., 1995; Borges et al., 2003; Shapiro et al., 2008; Zhao et al., 2018), such as TNF-α, IL-6, and IL-1, as well as complement factor 3 (Kettenmann et al., 2011; Waltl and Kalinke, 2022). These cytokines can induce seizures on their own or lower seizure threshold (Vezzani et al., 2008). Border-associated CAMs secrete chemokine ligand 2 (CCL2), which attracts CCR2-expressing circulating monocytes from the bloodstream. Within hours, the BBB becomes impacted and more permissive. Among the first cells to infiltrate the brain are innate immune cells, such as macrophages and neutrophil granulocytes (Ravizza et al., 2008; Zattoni et al., 2011; Fabene et al., 2013; Bröer et al., 2016). Inflammation persists and is still found in the chronic epileptic stage (Ravizza et al., 2008; Dubé et al., 2010; Filibian et al., 2012). Interestingly, this phenomenon is also found in non-SE epilepsy models, such as viral encephalitis-induced epilepsy (DePaula-Silva et al., 2021), and genetic models, for instance, genetic absence epilepsy (Akin et al., 2011) or myoclonus epilepsy (Tegelberg et al., 2012).

**Figure 1:**
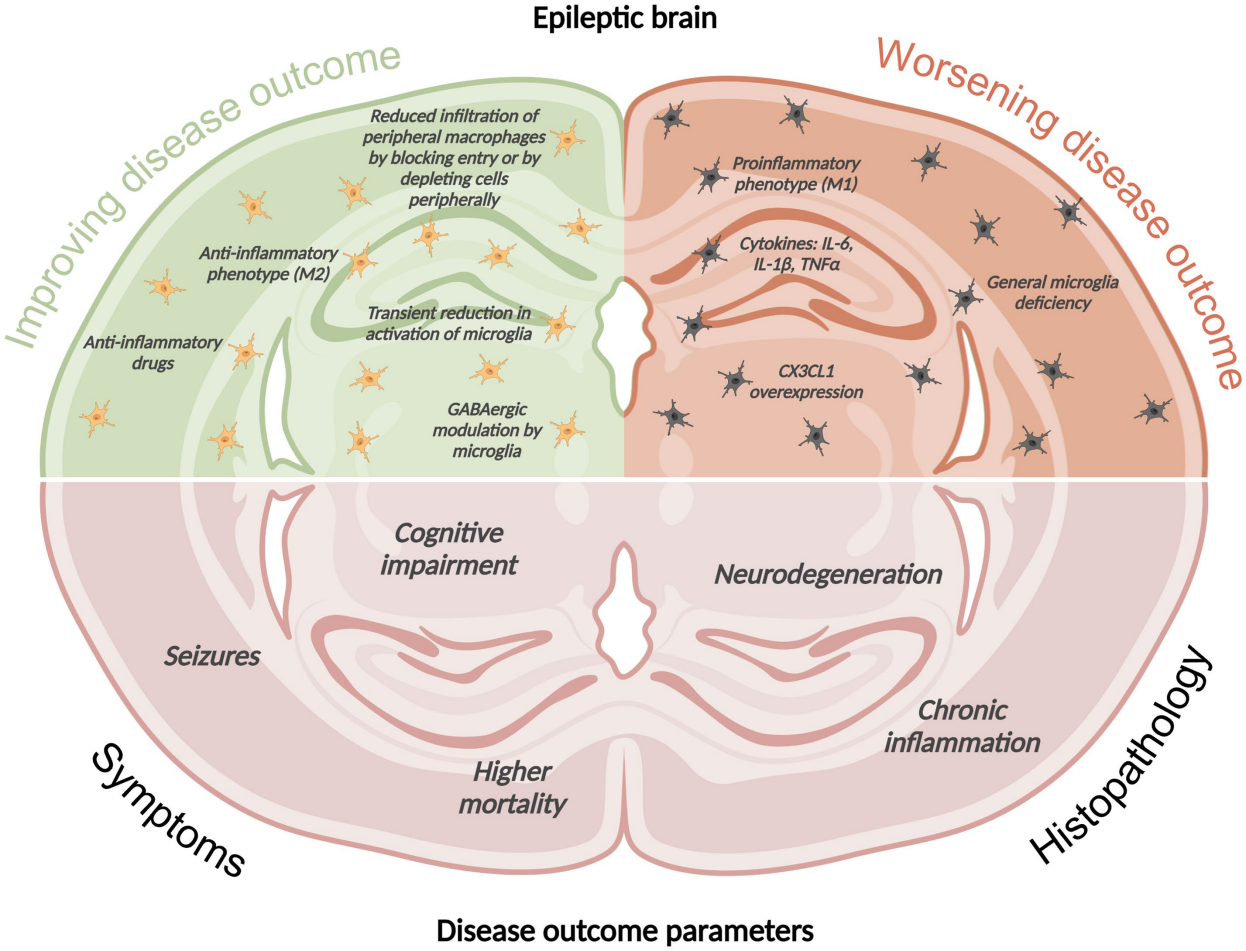
Simplistic overview of the current findings on the potential influence of phagocytes and their pro- and anti-inflammatory phenotypes on seizure activity, cognitive impairment, and brain pathology in experimental models of epilepsy. This image was created using BioRender.com.

We will focus on the role of resident microglia and infiltrating macrophages in seizure initiation and disease progression. Current literature points to a close interaction between microglia and macrophages, and a high plasticity in surface antigen expression that can make differentiation into either cell category quite challenging. What seems certain is that a delicate balance between pro- and anti-inflammatory monocytes is needed to combat infections and limit pathologies, such as seizures. A simplistic overview of the current findings on the potential influence of phagocytes and their pro- and anti-inflammatory phenotypes on seizure activity, cognitive impairment, and brain pathology in experimental models of epilepsy is depicted in Figure 1. The findings are discussed in depth in the following sections.

#### 3.3.1 Modulating phagocyte activity in seizure models

Some of the first studies investigating viral encephalitis-induced seizures in the Theiler’s Murine Encephalomyelitis Virus model (TMEV) confirmed that IL-6-producing cells are pivotal for seizure development (Kirkman et al., 2010; Libbey and Fujinami, 2011): Minocycline treatment as well as IL-6 deficiency modulated phagocyte activation and infiltration and reduced seizures (Cusick et al., 2013). Similarly, minocycline decreased microglial activation in a kainate-induced early-life SE model (Abraham et al., 2012). Treated mice were less susceptible to a second hit seizure in later life compared to controls (Abraham et al., 2012). Furthermore, if minocycline was applied before kainate-induced SE, apoptosis in the hippocampus was reduced (Heo et al., 2006). Minocycline treatment during the latent period reduced the later occurrence of chronic seizures (Wang et al., 2015). Of note, minocycline also inhibits the proliferation of other glia (Möller et al., 2016). Thus, the described effects cannot solely be attributed to phagocyte modulation.

Another successful strategy was to delete Apoptosis signal-regulating kinase 1 (ASK1) in microglia and macrophages because it is involved in inflammation and was upregulated in experimental animals and patients after seizures. The study revealed that ASK1^−/−^ reduced seizures, neurodegeneration, and cognitive impairment in the focal kainate mouse model (Zhang et al., 2022). Interestingly, ASK1 deletion modulated phagocyte polarization toward an anti-inflammatory phenotype (Zhang et al., 2022), while traditional SE models typically lead to early induction of M1 marker expression (Benson et al., 2015; Deng et al., 2020). Various other studies have shown that modulating microglia and macrophages toward M2 effectively alleviated disease outcomes (Liu et al., 2018, 2020; Li et al., 2023; Yang et al., 2024). Modern research has developed several tools, among which are cell-type specific genes and proteins, as well as reporter mice to distinguish pro- and anti-inflammatory monocytes, infiltrating, and resident monocytes. A recent review by Bosco et al. (2020) describes these technologies and methods very well.

#### 3.3.2 Modulating microglia in seizure models

Similar to human patients with TLE, induction of SE produced an overexpression of CX3CL1 and its receptor in rodents (Yeo et al., 2011). As a proof-of-concept, intracerebral infusions of CX3CL1 worsened neuronal damage after SE, while antibodies against CX3CL1 or its receptor alleviated it (Yeo et al., 2011). However, CX3CL1 treatment also modulated GABAergic function in *in vitro* studies on human TLE tissue (Roseti et al., 2013). By using a CX3CR1 deficient mouse, we reported that there was no difference in the accumulation of phagocytes in the CNS upon virus infection compared to WT mice. However, we found less pronounced activation and proliferation of phagocytes in CX3CR1^−/−^ mice (Käufer et al., 2018). In line with previous results, we found a neuroprotective effect, but no effect on seizures in the TMEV model (Käufer et al., 2018).

Other studies have evaluated the depletion of microglia to elucidate their role in epilepsy. Walt et al. used a pharmacological depletion approach by using PLX5622, a CSF1 inhibitor (2018b). They reported worse disease outcomes in the TMEV model, including higher virus persistence, increased mortality, earlier seizure occurrence, and increased inflammation, as well as neuronal damage (Waltl et al., 2018b), concluding that microglia are essential in fighting CNS infections and have a protective role (Waltl et al., 2018b). The time course of microglial involvement could be critical in determining their protective versus damaging potential, as Altmann et al. found that transient microglia depletion by a similar CSF1 inhibitor, PLX3397, during the early phase after SE induced by intra-amygdalar kainate injection was capable of preventing cell loss and cortical thinning. While the mice still developed epilepsy, they did not develop cognitive impairment (Altmann et al., 2022). More recent studies in the TMEV model proved that damage-sensing receptors such as P2YR12 undergo gene expression changes during acute infection and seizures (DePaula-Silva et al., 2019). Microglia became less responsive to damage signals during this vulnerable period (Wallis et al., 2024). Earlier work showed that seizure development depended on IL-6 secretion (Libbey et al., 2011) and that IL-6 was mainly derived from infiltrating macrophages (Cusick et al., 2013). By blocking macrophage invasion with an anti-inflammatory drug, seizure occurrence was reduced (Cusick et al., 2013). In addition, recent data from Howe’s lab suggested that while microglia activation could induce mild seizures and increase seizure susceptibility independent of IL-6 and TNF-α induction, microglial responses did not scale with the amount of virus inoculum, level of neuronal damage, seizure burden, or cognitive outcomes in the TMEV model (Howe et al., 2022).

#### 3.3.3 Modulating macrophages in seizure models

Macrophage entry across the BBB offers direct therapeutic interventions by either depleting peripheral macrophages or blocking their CNS entry. Zattoni et al. (2011) performed depletion studies in the intrahippocampal kainate mouse model by clodronate liposomes. CNS macrophage invasion was drastically reduced, leading to decreased granule cell dispersion, a hallmark of epileptogenesis in this model. In comparable studies across different labs, Waltl et al. (2018a) and DePaula-Silva et al. (2018) achieved a significant reduction of infiltrating macrophages after clodronate liposome treatment. They reported a decrease in acute seizures of 40–55% in the TMEV model. However, microglia activation within the brain did not seem to be altered, and neurodegeneration after virus infection was unchanged. Other groups have shown that the depletion of macrophages by the Gr1 antibody led to neuroprotective effects (Howe et al., 2012). Infiltrating monocytes were described to display a robust pro-inflammatory phenotype by expressing high levels of IL-1ß and MHCII 24 and 96 h after systemic pilocarpine injection in mice, while microglia did not (Vinet et al., 2016).

Another strategy to reduce macrophage invasion into the CNS is to block their entry by knocking out CCR2 (Tsou et al., 2007). CCR2^−/−^ mice recovered faster from systemic kainate SE (Varvel et al., 2016), experienced less severe seizures in the TMEV model (Käufer et al., 2018), and reduced recurrent seizures in a focally induced kainate model (Tian et al., 2017). In all three studies, neuronal damage was significantly reduced (Varvel et al., 2016; Tian et al., 2017; Käufer et al., 2018), and behavioral impairments were improved (Tian et al., 2017). Interestingly, CCR2 deficiency also reduced microglial proliferation upon infection compared to WT controls (Käufer et al., 2018), thus making clear that even rather specific genetic targeting approaches might still influence other (immune) cell populations.

## 4 Conclusion

While our knowledge about phagocyte biology in brain homeostasis has exponentially grown, and evidence of their involvement in various CNS pathologies is increasing, a lot remains to be learned. It can convincingly be shown that epileptogenesis, epilepsy, and seizures are closely linked to CNS inflammation [cf. Vezzani (2014)]. However, there is no clear causal relationship: Inflammation facilitates seizures, while seizures also produce inflammatory reactions in the brain (Vezzani et al., 2011). Comparing DNA-methylation and gene expression alterations in neurons and glia as upstream mechanisms of experimental epileptogenesis, Berger et al. (2019, 2020). However, we still cannot fully determine the contributions of these cells to resolving, maintaining, or even worsening inflammatory conditions and their long-term outcomes on CNS health. Many of the reported findings have been reproduced across various models of seizures and epilepsy. Resident microglia and infiltrating macrophages can either act pro- or antiepileptogenic depending on their distinctive phenotype, activation pattern, duration, and time point. Microglia activation in acute disease states such as infection-induced seizures is crucial for pathogen elimination and host survival, as well as protective for neurons and inhibitory networks. On the contrary, pro-inflammatory macrophages mostly appear to be detrimental during this early phase. Future technologies will enable us to further dissect the function of these cell types and their plastic phenotypes during chronic and progressive neurological diseases such as epilepsy. Understanding the roles of phagocytes in ictogenesis and the time course of their activation and involvement in epileptogenesis can offer us new biomarkers to identify patients at risk, as well as provide novel therapeutic targets. The challenge lies in developing targeted therapies that modulate their activity to harness beneficial effects while minimizing harmful outcomes.

## Author contributions

SB: Writing – review & editing, Writing – original draft. AP: Writing – original draft.
